# Voltage and Deflection Amplification via Double Resonance Excitation in a Cantilever Microstructure

**DOI:** 10.3390/s19020380

**Published:** 2019-01-18

**Authors:** Mohammad H. Hasan, Fadi Alsaleem, Abdallah Ramini

**Affiliations:** 1 The Mechanical Engineering Department, University of Nebraska at Lincoln, Lincoln, NE 68588, USA; mohammadhhasan@huskers.unl.edu; 2 The School of Architectural Engineering University of Nebraska at Lincoln, Omaha, NE 68182, USA; 3 The Mechanical Engineering & Mechanical Engineering Technology, Penn State Harrisburg, Harrisburg, PA 17057, USA; aur1191@psu.edu

**Keywords:** double-resonance, cantilever microstructure, RLC circuit

## Abstract

Cantilever electrostatically-actuated resonators show great promise in sensing and actuating applications. However, the electrostatic actuation suffers from high-voltage actuation requirements and high noise low-amplitude signal-outputs which limit its applications. Here, we introduce a mixed-frequency signal for a cantilever-based resonator that triggers its mechanical and electrical resonances simultaneously, to overcome these limitations. A single linear RLC circuit cannot completely capture the response of the resonator under double resonance excitation. Therefore, we develop a coupled mechanical and electrical mathematical linearized model at different operation frequencies and validate this model experimentally. The double-resonance excitation results in a 21 times amplification of the voltage across the resonator and 31 times amplitude amplification over classical excitation schemes. This intensive experimental study showed a great potential of double resonance excitation providing a high amplitude amplification and maintaining the linearity of the system when the parasitic capacitance is maintained low.

## 1. Introduction

In the age of Internet of Things (IoT), data is collected at an increasing rate. Microelectromechanical systems (MEMS) devices are considered as prime candidates for IoT sensors. Currently, MEMS devices are used as high-frequency switches [1,2], sensors [3,4,5,6,7,8], RF amplifiers [9], mirrors, [10], and oscillators [11,12,13]. These developments led to fabricate and integrate MEMS devices into silicon chips easily, and achieve the requirements of low power, low cost, and smaller weight.

Electrostatic actuation is a popular choice for MEMS devices. It offers advantages such as high sensitivity and low floor noise compared to other MEMS devices [14]. However, electrostatic MEMS requires high actuation voltage [15,16], limiting its implementation in many applications and systems. Moreover, the deflection of MEMS is often limited to small amounts due to the risk of pull-in (collapse of the microbeam into the substrate due to electrostatic force) [17]. Therefore, many methods were proposed to reduce the input voltage requirement by decreasing the air gap between the actuation electrodes, decreasing the MEMS structural stiffness, or increasing the electrostatic actuation area [18,19,20]. However, most of these methods limit the MEMS operations because they increase the undesirable squeeze film damping effect [21], high noise outputs, the collapse of these structures, or the vulnerability of these structures to stiction [22]. Furthermore, decreasing the MEMS structure’s stiffness leaves it more vulnerable to shock.

Utilizing mechanical resonance is a common way of amplifying the response of a MEMS structure. Previous works extended this concept using multifrequency excitation signals to increase MEMS filter bandwidth [23,24,25,26], the signal-to-noise ratio in microgyroscope applications, [27] and the harvested energy in MEMS harvester [28]. Finally, as MEMS devices also act as capacitors; electrical resonance was utilized for detection through electrical resonant frequency shift in an RLC circuit [29] and to amplify the MEMS response by forming an LC tank circuit or a resonant drive circuit [30,31]. Triggering electrical resonance in such a circuit leads to a large voltage amplification across the MEMS device. However, due to the mismatch between the mechanical and electrical resonances, only static amplification can be achieved using a typical excitation signal. Dynamic amplification is possible using more complex driving signals, such as frequency or amplitude modulated signals [32,33].

In a recent work, we demonstrated experimentally double resonance excitation [33] as a way to achieve dynamic amplification in a clamped-clamped MEMS device. Double resonance occurs when exciting the MEMS device, within an RLC circuit, at the electrical and mechanical resonances, simultaneously, using a simple mixed frequency signal. In this work, we extend the double resonance excitation concept for a cantilever-based MEMS device. We also, for the first time, build a mathematical model to describe the interaction between the mechanical and electrical components of the system, under double resonance excitation and validate this model with experimental data. Furthermore, we explore the use of the developed model to show the high potential of double resonance excitation in cantilever microbeams.

The paper organization is as follows. In Section 2, we present the cantilever-based microbeam (mechanical and electrical) models and the experimental setup followed by the results in Section 3. Finally, we discuss our conclusions in Section 4.

## 2. Device and System Modeling

**Figure 1:**
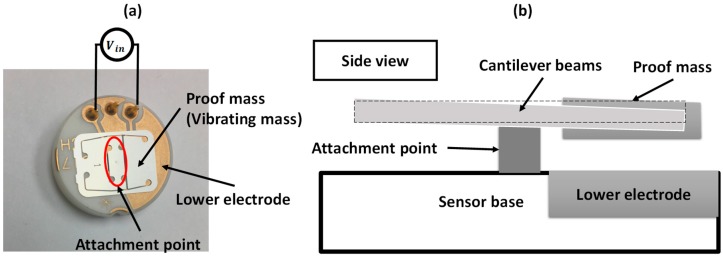
The device used in this study. (**a**) A picture top view. (**b**) A schematic of the side view.

**Figure 2:**
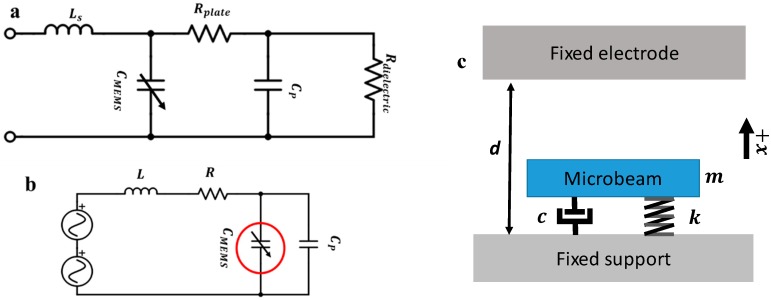
The microelectromechanical systems (MEMS) device electrical and mechanical model schematics. (**a**) A schematic for the MEMS equivalent circuit. The resonator is modeled as an imperfect capacitance with a small series (lead) inductance (L_s_) and a variable capacitance (C_MEMS_) reflecting the change in capacitance because of the motion, parallel parasitic capacitance (C_p_), a negligible plate resistance (R_plate_), and an almost infinite parallel dielectric resistance (R_dielectric_). All components aside from C_MEMS_ and C_p_ are negligible in the model. (**b**) A simplified RLC circuit model for the MEMS circuit consisting of the resonator and the externally added resistance R and inductance L. (**c**) Single-degree-of-freedom mechanical model of the resonator.

Resonators are electromechanical components; therefore, we model them mechanically and electrically. A Sensata™ MEMS accelerometer [34] consisting of an out-of-plane-displacement, electrostatic double-cantilever (a paddled structure) was used as the experimental design. A schematic of the device is shown in Figure 1. Figure 2 shows schematics for the MEMS electrical and mechanical models. The full electrical and mechanical model equations of the device are explained in detail in the Supplementary Materials. The mechanical parameters of the structure were extracted in previous work [17] and can be found in Table 1. We note here that the MEMS deflection (motion) may change the electrical characteristics of the MEMS device. Therefore, the MEMS device is typically modeled as a variable capacitance (CMEMS) connected to a motional inductance and a motional resistance. In this work, however, the motional inductance and resistance values are negligible as the MEMS is connected to an external inductance with high internal resistance value.

MEMS devices are often used as resonators by exciting them at their mechanical resonance frequency to increase the sensitivity to the force. As the voltage amplification translates into force amplification, it is desirable to operate the resonator at the mechanical resonance and the electrical resonance simultaneously. However, these resonances are rarely equal, with the electrical resonance being typically much higher than the mechanical resonance frequency of a MEMS device. One way to solve this problem is to control the external inductance of the system [33]. However, this method is viable only if the difference between the two resonances is small.

Moreover, increasing the inductance results in a higher parasitic resistance in the circuit, and hence, a lower voltage gain is obtained as shown in Equation (S12). Another means to solve this issue is using a mixed-frequency signal to trigger the two resonances simultaneously. This concept is demonstrated in the next subsection.

### 2.1. Double Resonance Excitation

The mechanical resonance of a MEMS device is typically excited using single DC and AC sources. However, to activate two dissimilar resonances simultaneously, a different approach that includes two AC sources is used in this work. By analyzing Equations (S7) and (S8), the forcing term of the mechanical system is a quadratic function, while the forcing term of the electrical system is a linear function. While it is possible to activate the electrical resonance using a signal with a frequency component matching the electrical resonance frequency, to simultaneously activate the mechanical resonance, an input voltage signal composed of two frequency components is proposed:

(1) $$V=V_{AC1}\cos2{\pi f}_{1}t+V_{AC2}\cos2{\pi f}_{2}t$$

Substituting (1) into (S7) yields (2).

(2) $$\begin{array}{r} F_{e}(V)=\frac{\varepsilon A_{s}}{2{\left(d-x\right)}^{2}}(\frac{V_{AC1}^{2}}{2}[1+\cos(4{\pi f}_{1}t)]+\frac{V_{AC2}^{2}}{2}[1+\cos(4{\pi f}_{2}t)]+V_{AC1}V_{AC2}[\cos\{2{\pi (f}_{1}-f_{2})t\}+\\ \cos\{2{\pi (f}_{1}+f_{2})t\}]) \end{array}$$

Therefore, the frequency components of the force term acting on the mechanical system are 2*f*_1_, 2*f*_2_, *f*_1_ − *f*_2_, *f*_1_ + *f*_2_ as well as the DC component. Any one of these frequency components activates the mechanical resonance when it equals the MEMS primary resonance frequency. Therefore, using a mixed-frequency signal with one component equal to the electrical resonance frequency while the other frequency results in any of the forcing frequency combinations equaling the mechanical resonance frequency will activate both resonances simultaneously. Here, the tested resonator has a very low mechanical resonance frequency that is far smaller than the electrical resonance frequency. Thus, the proper frequency component to be excited is *f*_1_ − *f*_2_, which allows both *f*_1_ and *f*_2_ to activate the electrical resonance.

### 2.2. Experimental Setup

**Figure 3:**
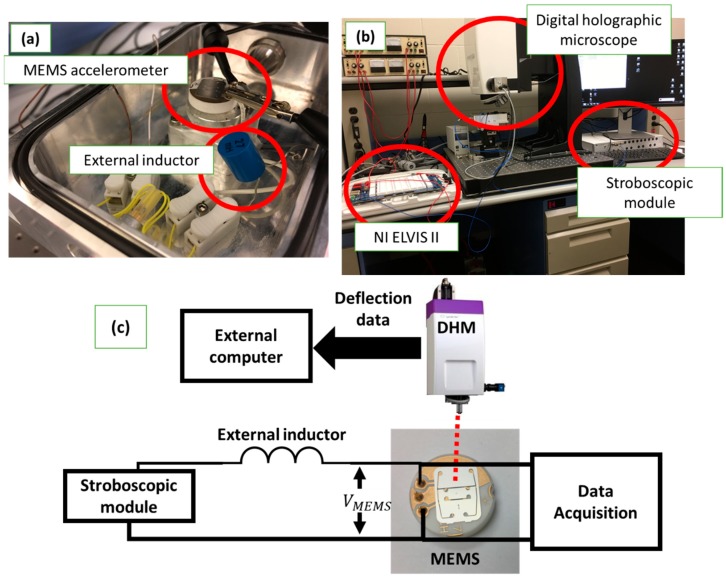
Experimental setup. (**a**) Circuit connection showing the accelerometer and the external inductor. (**b**) The equipment used for measurements: NI ELVIS II, DHM, and stroboscopic module. (**c**) The connection schematic.

Using Lyncee Tec’s digital holographic microscope (DHM), we measure the out-of-plane displacement that corresponds to the mechanical response of the resonator in the experimental setup shown in Figure 3. The stroboscopic module drives the circuit, composed of the resonator and an external inductor. We measured the input signal and the voltage across the resonator through different virtual channels in the NI ELVIS II module.

## 3. Results

### 3.1. Electrical Circuit Identification

**Figure 4:**
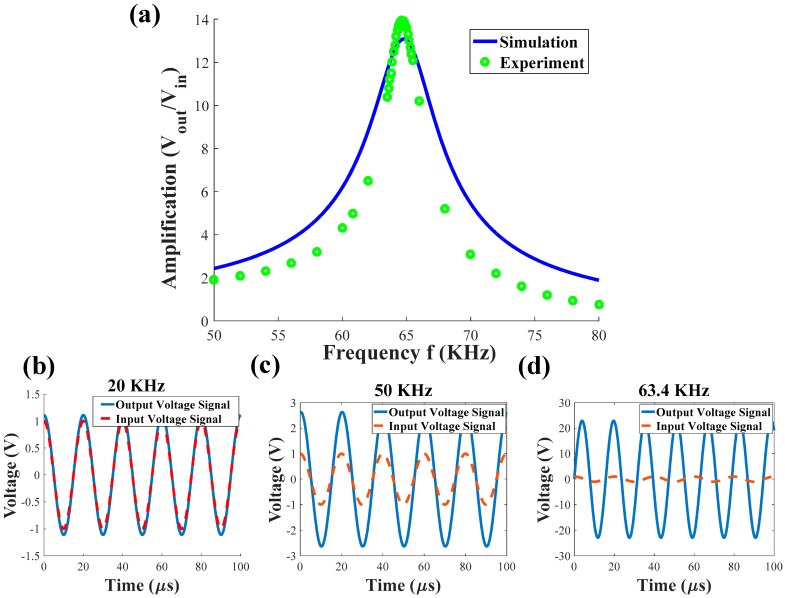
Theoretical and experimental study of the electrical circuit identification and the electrical resonance of the MEMS device in the resonance RLC circuit. (**a**) Circuit frequency response is showing the amplification (gain) of the voltage across the capacitance. The green circles show the experimental voltage across the MEMS device while the solid blue line shows the model simulation results. The electrical resonant frequency and voltage amplification were found to be 64.6 kHz and 13 times, respectively. Furthermore, the time history of the input signal and the output signal at different input frequency values are shown in (**b**–**d**). A phase shift is observed between the input signal and the output signal at 63.4 kHz, which is a characteristic of the electrical resonance of the system.

In the beginning, the device is driven in the air (1 atm) and with a low input voltage to reduce the vibration and the transient deflection of the resonator; therefore, reducing the structure to a constant capacitance and measuring the gain of the circuit shown in Figure 4. The electrical resonance frequency, corresponding to a gain of 13 times, is found to be 64.6 kHz. Using the nonlinear fitting tool in MATLAB, we fitted the experimental data into the model of the series RLC circuit using Equation (S12). Table 2 shows the circuit parameters of the used RLC circuit. The nominal capacitance was computed using (S15), while the parasitic capacitance and the resistance were estimated using a curve fitting technique. While the MEMS circuit is more complex than a simple RLC circuit, the fitted curve captures the general electrical behavior of the circuit. A more advanced electrical model is left to a future study.

### 3.2. Double Resonance as a Driving Signal

**Figure 5:**
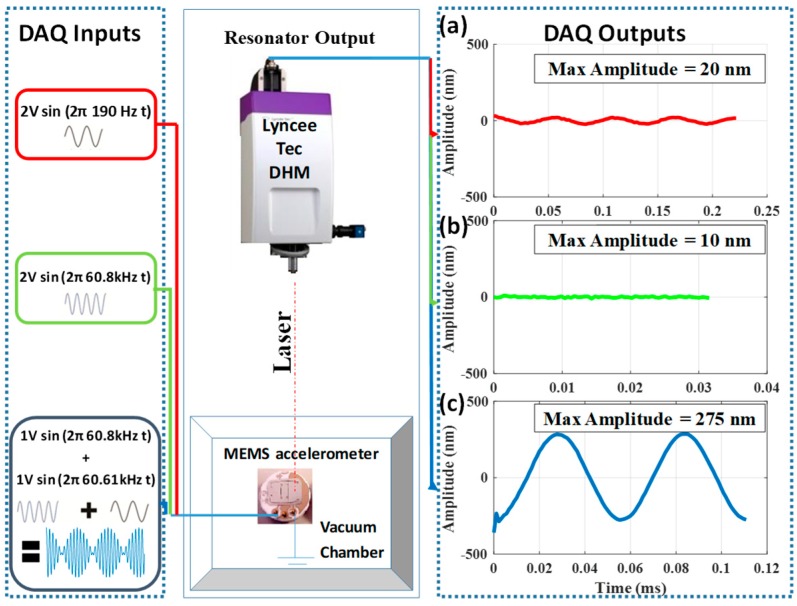
The experimental out-of-plane deflection of the resonator for three different cases. (**a**) Close to the mechanical resonance frequency, a sinusoidal signal of 190 Hz and 2 V of amplitude generates a deflection amplitude of 20 nm. (**b**) Relatively close to the electrical resonance of the circuit, a sinusoidal signal of 60.8 kHz and 2 V of amplitude generates a deflection amplitude of ~10 nm (within noise of the measurement). (**c**) A mixed signal composed of two frequencies: 60.8 kHz and 60.61 kHz, each with 1 V of amplitude, generates a deflection amplitude of 275 nm.

In this section, we compare the response of the resonator for the following excitation cases; mechanical resonance only, electrical resonance only, and double resonance. In this comparison, the ambient pressure was reduced to around 20 Pa to overcome the high viscous damping. First, the accelerometer was excited near its mechanical resonance, using a 2 V AC-amplitude signal of a frequency of 190 Hz. Consequently, the amplitude of the out-of-plane deflection reaches around 20 nm as shown in Figure 5a. Next, we excited the accelerometer by using a 2 V AC-amplitude signal of 60.8 kHz near the electrical resonance frequency. Due to electrical resonance activation, the input voltage was amplified up to five times. However, the resonator attenuates the effect of the voltage amplification and reduces the deflection to 10 nm as the AC excitation frequency gets far from the mechanical resonance frequency (Figure 5b). Therefore, to trigger both voltage and mechanical amplification, an enhanced signal that excites the high-frequency electrical resonance and low-frequency mechanical frequency simultaneously is needed. To overcome this dilemma, we use a signal with two appropriate frequency components (a beating signal): One component of the proposed AC excitation signal is chosen to be near the electrical resonance, while the absolute difference between the two frequency components should be near the mechanical resonance magnitude. We experimentally obtained this signal by driving the resonator with two AC signals of 60.8 kHz and 60.61 kHz. The resonator responds to the difference between the two signals (190 Hz), while the electrical circuit amplified the two input components. The use of the mixed-frequency signal results in a large vibration amplitude of 275 nm (Figure 5c). Therefore, we show that we can use a small AC actuation voltage to trigger a large mechanical deflection of the resonator while simultaneously amplifying the voltage across it through double resonance excitation.

### 3.3. Frequency Sweep When Driven Using Mixed-Frequency

**Figure 6:**
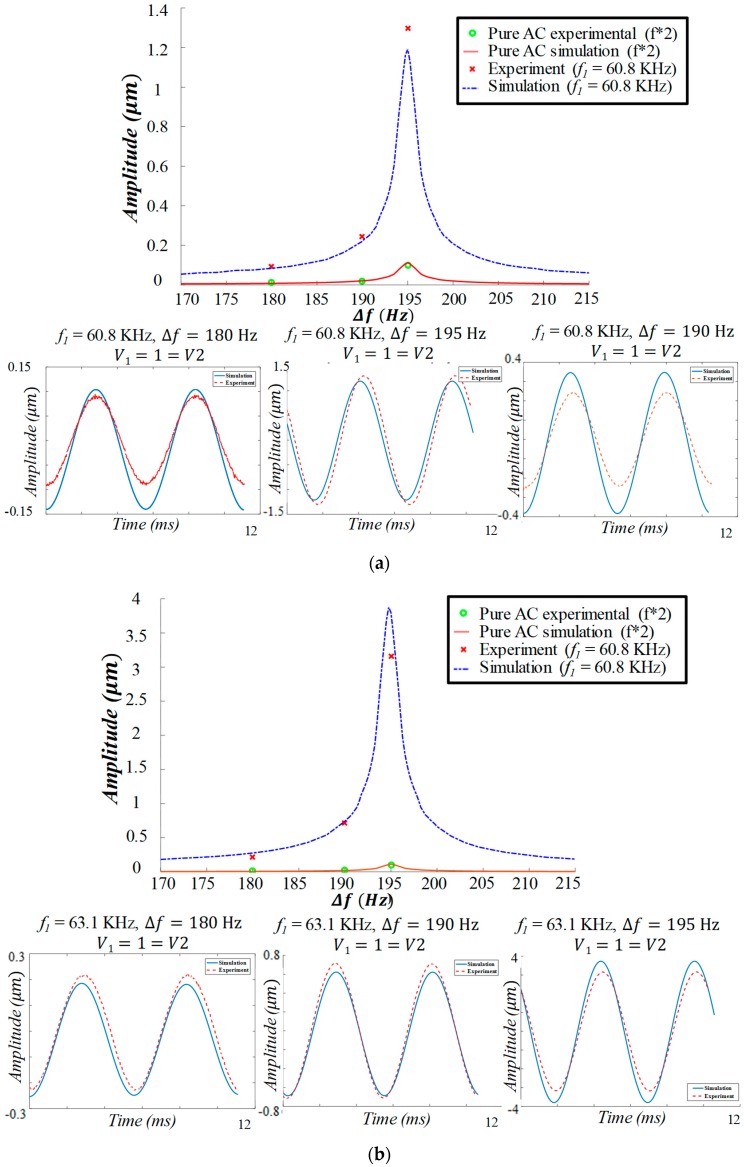
Double resonance excitation using a beating signal composed of two voltage sources each with an amplitude of 1 V: (**a**) a frequency of *f*_1_ = 60.8 kHz and (**b**) a frequency of *f*_1_ = 63.1 kHz and *f*_2_ is swept both cases. Experimental results are shown with crosses and simulation is shown by a dashed line. Both cases are compared to the experimental and numerical simulation frequency response when excited classically by a single AC source at a frequency range of 85–107.5 Hz at 2 V, which has a maximum amplitude of 0.1 μm. This is denoted by the label “2*f*” to indicate that the effective frequency due to a single AC source is double the input frequency (frequency doubling).

As the proposed new input signal has two frequency components, we perform frequency sweep by fixing one of the excitation frequencies (*f*_1_) while sweeping the other frequency (*f*_2_). Figure 6a,b shows experimentally and numerically the frequency response and time history response of the resonator when driven with *f*_1_ = 60.8 kHz and *f*_1_ = 63.1 kHz, respectively, while sweeping *f*_2_. Each frequency component has an amplitude of 1 V_AC_. In both cases, the responses of the resonator are compared to the response of a single sinusoidal input force around the mechanical resonance with an amplitude of 2 V as a reference. The pure AC signal is swept from 85 Hz to 107.5 Hz. The input frequency was chosen to be swept at approximately half the MEMS mechanical resonance frequency because of the frequency doubling. In this case, the MEMS device vibrates at double the input frequency.

While driven by a single AC source, the resonator has a maximum amplitude of 0.1 µm at 97.5 Hz. However, the resonator has a higher maximum amplitude of 1.3 µm with 13 times amplification (Figure 6a) and 3.16 µm at 195 Hz with 30 times amplification (Figure 6b) while driven by a double resonance excitation. Higher amplitude is shown when *f*_1_ = 63.1 kHz because it is closer to the electrical resonance frequency of the circuit compared to *f*_1_ = 60.8 kHz. Despite the high amplitude, the response of the resonator is like that of a linear resonator.

Next, we compare the obtained experimental data to the simulation results obtained by solving (S1) and (S8) simultaneously, accounting for the capacitance change due to the MEMS deflection and squeeze film damping (S6). The solution is obtained numerically using the Runge–Kutta method assuming zero initial conditions. We note here that the global electrical fitted model of Section 3.1 in Figure 4 does not converge quite as well with the attained experimental data of the MEMS deflection. This imperfection might be because of the added components involved in the circuit (BNC cable between the stroboscopic model and the RLC circuit and the data acquisition system) or the additional parasitic in the circuit influencing the behavior of the RLC circuit more significantly at higher deflections. To overcome this challenge, we preformed localized fitting of the circuit parameters around *f*_1_ = 60.8 kHz and *f*_1_ = 63.1 kHz. The simulated response, for both cases, is shown in Figure 6 with dashed lines. The simulations with the local fits appear to match the experimental data very closely.

**Figure 7:**
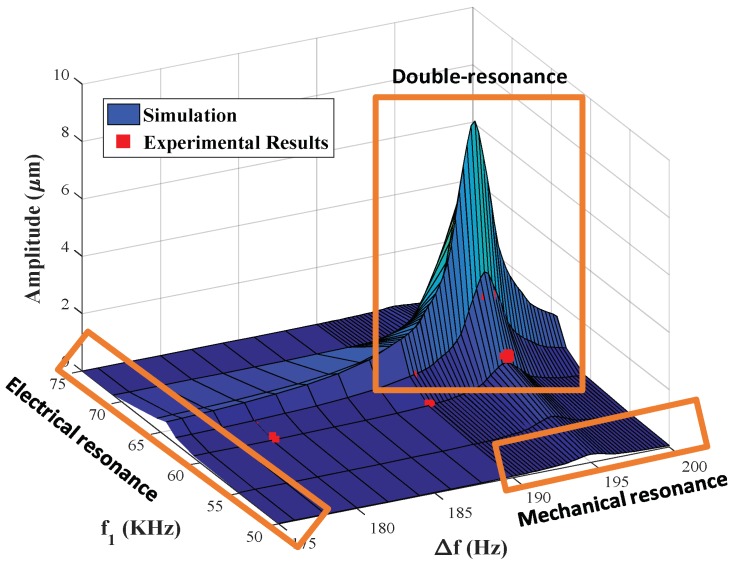
Three-dimensional plot of the simulated frequency response as a function of the excitation frequencies *f*_1_ and (*f*_2_ = *f*_1_ + Δ*f*). The voltage of each signal component is *V*_1_ = *V*_2_ = 1 V. The experimentally obtained points are in red.

Next, we construct a three-dimensional plot of the mechanical response of the resonator as a function of the two excitation frequencies (*f*_1_ and *f*_2_), with frequency components of *V_AC_* = 1 V each in Figure 7. However, we replace *f*_2_ with Δ*f* for the sake of clarity and to simplify the identification of the mechanical resonance frequency. Figure 7 shows relative maxima around *f*_1_ = 64.6 kHz and Δ*f* = 195 Hz, which correspond to the electrical resonance frequency and the mechanical resonance frequency, respectively. Regardless of the value of *f*_1_, the electrical and the mechanical resonances can individually amplify the input signal even without interacting with each other. Moreover, a higher vibrational amplitude equals 8.9 μm at *f*_1_ = 64.6 kHz and Δ*f* = 195 Hz, which corresponds to the electrical circuit resonance and the mechanical resonance, respectively, showing the constructive multiplicative effects of electric and mechanical amplification. This makes double-resonance excitation a powerful excitation method that can be used to actuate any MEMS or NEMS device with no regards to the internal design of the device and without changing the overall response behavior of the resonator.

The existence of a large amplitude regime in the 3-D plot in Figure 7 is formed by the intersection of the electrical resonance regime and the mechanical resonance regime. This high amplitude far exceeds the individual contribution of either resonance. Classical actuation can only access this behavior means if the resonator is carefully designed to have an electrical resonance frequency close to the mechanical resonance frequency. While this imposes tight design tolerances, such as the need to greatly control the circuit parasitic, it remains possible. However, without changing the actual mechanical design, we showed using a double-resonance to trigger the two primary resonance frequencies of the systems simultaneously. Therefore, the actual voltage requirements are significantly lowered due to the large amplification of the signal.

Moreover, in this figure, the MEMS response retains linearity due to the electromechanical capacitance coupling. Hence, its electrical resonance frequency is a function of the MEMS deflection. When the MEMS deflection increases, the electrical resonance frequency shifts and the electrical gain decreases, thus reducing any further MEMS deflection. Therefore, the system possesses inherent negative feedback when actuated using double resonance excitation. This negative feedback is more noticeable when the ratio between the parasitic capacitance and the MEMS nominal capacitance is relatively small.

**Figure 8:**
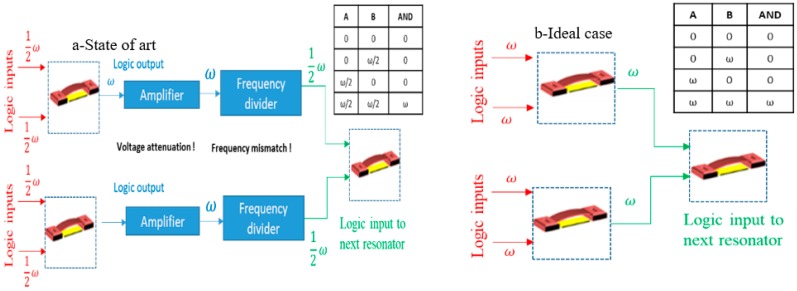
The MEMS cascaded and logic gate. (**a**) The need to use MEMS as the next logic gate led to the proposition of adding very complex complementary metal-oxide-semiconductor (CMOS) chips to enable the cascading MEMS-based logic gate. (**b**) Ideally, we want to achieve CMOS functionalities using MEMS only.

Finally, this operation concept could potentially enable MEMS to be the next logic gate. While considerable attention has been given to MEMS resonators to serve as the next logic gate chips due to their low power consumption and high integration density capabilities [2], none of the reported methods in the literature allows cascading multiple MEMS logic gates since they require different input and output signals and because the output signals suffer from huge voltage attenuation. A state-of-the-art solution proposed for these problems is to place amplifiers and frequency dividers between MEMS logic gates to function [26] in Figure 8a. Ideally, we want a MEMS logic gate resonator that can be easily integrated with other logic gates to form a complete integrated circuit (IC) without the need for any complementary metal-oxide-semiconductor (CMOS) part, Figure 8b. The high voltage amplification due to double resonance activation presented in this work could eliminate the need for amplifiers in MEMS logic gates. Moreover, the need for frequency dividers could be eliminated by activating subharmonic resonance. At subharmonic resonance, the MEMS vibrates at a frequency near its fundamental natural frequency (half the input signal frequency) [17]. 

## 4. Conclusions

In summary, we expand the concept of double resonance excitation using a mixed-frequency signal by providing more experimental results and a mathematical model validated from these results. The mathematical model of the circuit was shown to be overly simplistic as it does not account for the parasitic components arising from the electrical connections and the possible change in the parasitic capacitance due to the change in gap. However, this was alleviated by fitting the electrical frequency response to a simple series RLC circuit response at the frequency of interest. Here, we showed the electromechanical model was able to predict the large amplitude amplification due to double resonance in this model. Furthermore, we showed the great potential of double resonance excitation providing a high amplitude amplification.

Finally, as energy is conserved in this system, the double resonance amplitude gain is achieved because of the increase of current drawn from the source, and hence, the input power increases by the reduction in the impedance of the system at electrical resonance. Thus, double resonance excitation trades at higher vibrational amplitudes and at lower input voltage with a high current and increased input power. However, as MEMS devices inherently require low input power, this power increase is expected to be acceptable for MEMS applications.

## Figures and Tables

**Table 1:** Mechanical properties of the structure.

Variable	Definition	Value
*Le*	Length of beam	9 mm
*b*	Width of beam	5.32 mm
*h*	Thickness of beam	150 μm
*d*	Nominal gap	42 μm
*k*	Linear stiffness	215 N/m
*f_m_*	The primary mechanical resonance frequency	195 Hz

**Table 2:** The electrical properties of the resonator.

Variable	Definition	Value
*L*	Total circuit series inductance	25.66 mH
*R*	Total circuit series resistance	799.2 Ω
*C*_0_	Nominal capacitance	10 pF
*C_p_*	Parasitic circuit capacitance	224.74 pF

## Supplementary Materials

The following are available online at http://www.mdpi.com/1424-8220/19/2/380/s1.
