# Supplementary material for: Voltage and Deflection Amplification via Double Resonance Excitation in a Cantilever Microstructure
Source: Sensors (Basel). 2019 Jan 18;19(2):380. doi: 10.3390/s19020380 (PMC6359633; doi:10.3390/s19020380)
Supplement: Supplementary file 1 [file sensors-19-00380-s001.pdf]

sensors

MDPI

Supplementary Materials

Voltage and Deflection Amplification Via Double Resonance Excitation in a Cantilever Microstructure

Mohammad H Hasan [1], Fadi Alsaleem [2] and Abdallah Ramini [3,*]

[1] The Mechanical Engineering Department, the University of Nebraska at Lincoln, Omaha, NE, USA
[2] The School of Architectural Engineering University of Nebraska at Lincoln, Omaha, NE, USA
[3] The Mechanical Engineering & Mechanical Engineering Technology, Penn State Harrisburg, Harrisburg, PA, USA
* Correspondence: falsaleem2@unl.edu Tel.: +1-316-719-0450

1. The device as a mechanical resonator:

In the beginning, we model a MEMS resonator as a Single-Degree-of-Freedom (SDOF) spring-mass-damper system with linear stiffness (k) and nonlinear damping ($c(x)$). The deflection of the electrostatically-actuated resonator is

$$m_{eq}\ddot{x} + c(x)\dot{x} + kx = F_e(x) \tag{S1}$$

where x is the deflection of the tip of the cantilever, positive towards the substrate beneath the microbeam, and the dot operator represents derivation with respect to time t, k is the linear stiffness of the microbeam. For a double cantilever, $k = 2 \times 3EI/L^3$ where E is the Young modulus of elasticity, I is the second moment of area of the beams and L is the beam length. $m_{eq} = k/\omega_n^2$ is the equivalent mass of the microbeam, $c(x)$ is the nonlinear squeeze film damping and F_e is the electrostatic force acting on the microbeam. The nonlinear squeeze film damping is solved by (S2-S6) sequentially [1, 2]:

$$\lambda_a = \frac{\lambda_0 P_0}{P_a} \tag{S2}$$

$$Kn = \frac{\lambda_a}{d} \tag{S3}$$

$$\mu_{eff} = \frac{\mu}{1+9.638Kn^{1.159}} \tag{S4}$$

$$\sigma(x) = \frac{12A_s(2\pi f_m)\mu_{eff}}{P_a(d-x)^2} \tag{S5}$$

$$c(x) = \frac{64\sigma(x)P_aA_s}{\pi^6(2\pi f_m)(d-x)}\frac{(1+\beta^2)}{\left[(1+\beta^2)^2+\frac{\sigma(x)^2}{\pi^4}\right]} \tag{S6}$$

where P_a is the ambient (operation) pressure, λ_a , λ_0 are the mean-free path of gas molecules at the operating pressure and atmospheric pressure, respectively, K_n is the Knudsen number, μ is the nominal dynamic viscosity constant of air and μ_{eff} is the effective viscosity constant of air to account for the slip boundary condition, $\beta = b/L$ is the shape ratio, which is the ratio between the width of the microbeam b and length L, f_m represents the mechanical resonance frequency of the device, $A_s = b\,L$ is the area of overlap between the microbeam proof mass and the substrate beneath it, d is the nominal separation distance between the microbeam and the substrate, and σ(x) is the squeeze number. We demonstrated in a previous work that modeling squeeze film damping as the only source of damping yields to adequate results [3]. Thus, other forms of damping are neglected.

The electrostatic force [3] is given by (S7):

$$F_e = \frac{\varepsilon A_s(V_{DC}+V_{AC}\ \cos(2\pi ft-\emptyset))^2}{2(d-x)^2} \tag{S7}$$

where V_{DC} is the DC voltage and V_{AC} is the AC voltage across the resonator, ϕ is the AC phase shift, and $\varepsilon = \varepsilon_0\ \varepsilon_r$ is the permittivity of the dielectric separating the electrodes, ε_0 is the permittivity of

vacuum, and ε_r is the relative permittivity of the dielectric. As most simulations and experiments are taken either in vacuum or relatively dry air, ε_r is set to unity.

2. The device as an electrical resonator:

Resonators are classically modeled electrically as ideal lumped capacitive elements. These models are valid for operational frequencies significantly lower than the electrical resonance frequency of the circuit. Thus, researchers limit the operational range of frequencies to lower than the electrical resonance.

To extend the range of the electrical model, one must consider the parasitic components of the resonator (Inductance – L_s, Resistance – [$R_{dielectric}$, R_{plate}, Re_{wires} (very small)] and Capacitance - C_p) in the model. C_{MEMS} is the variable capacitance and is the sensing element of the circuit. We note that the series R_{plate} and R_{wires} are very small and are neglected in a circuit with external series resistance. Moreover, the parallel $R_{dielectric}$ is very large and can be assumed an open circuit if the applied voltage is smaller than the breakdown voltage of the material. Therefore, we only consider these parasitic components L_s and C_p and the simplified circuit in this study, as shown in Figure S1. This consideration results in a series RLC circuit govern by (S8):

$$L\ \ddot{Q}(t) + R\ \dot{Q}(t) + \frac{1}{C}\ Q(t) = V_{in}(t) \quad \text{(S8)}$$

where $L=L_s+L_{external}$ is the total series inductance, equal to the parasitic series inductance plus any external inductance, $R=R_L+R_{external}$ is the total external series resistance and equals to the parasitic resistance of the inductor plus any external resistance, C is the total series capacitance given by (S9), $Q(t)$ is the charge stored in the capacitance and $V_{in}(t)$ is the input voltage.

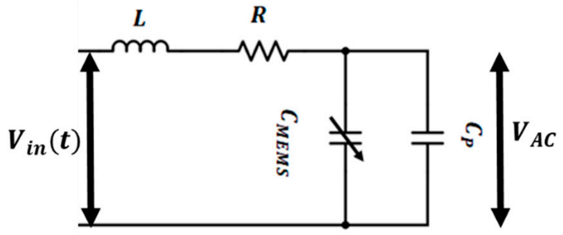

Figure S1. MEMS circuit schematics

$$C = C_p + C_0 = C_p + \frac{\varepsilon A_s}{d} \quad \text{(S9)}$$

where C_0 is the nominal capacitance of the MEMS. By studying the total impedance of the circuit, we find that:

$$Z_{eq} = R + X = R + \frac{j((2\pi f)^2 LC - 1)}{(2\pi f)C} = \frac{(2\pi f)RC + j((2\pi f)^2 LC - 1)}{(2\pi f)C} \quad \text{(S10)}$$

where Z_{eq} is the equivalent impedance of the circuit, X is the equivalent reactance of the circuit, f is the AC frequency and j is the imaginary number. Furthermore, by studying the voltage across the capacitance, either by solving (S8) or by voltage division yields:

$$V_{AC} = |V_{AC}| \cos(2\pi f t - \phi) \quad \text{(S11)}$$

$$\left|\frac{V_{AC}}{V_{in}}\right| = \frac{1}{\sqrt{((2\pi f)RC)^2 + ((2\pi f)^2 LC - 1)^2}} \quad \text{(S12)}$$

$$\phi = \tan^{-1}\left(\frac{(2\pi f)^2 LC - 1}{(2\pi f)RC}\right) \quad \text{(S13)}$$

Equation (S12) shows a voltage amplification with a maximum at the electrical resonance frequency (f_e) of the RLC circuit.

$$f_e = \frac{1}{2\pi\sqrt{LC}} \quad \text{(S14)}$$

Finally, as the gap changes because of the electrostatic forcing, the actual capacitance of the system varies and is given by:

$$C_0 = \frac{\varepsilon A_s}{d} \tag{S15}$$

$$C(x) = C_p + C_0/(1 - x/d) \tag{S16}$$

and this value is used to replace *C(x)* in all previous equations.

References

1. Blech, J. J. (1983). On isothermal squeeze films. Journal of lubrication technology, 615-620.
2. Veijola, T., Kuisma, H., Lahdenperä, J., & Ryhänen, T. (1995). Equivalent-circuit model of the squeezed gas film in a silicon accelerometer. Sensors and Actuators A: Physical, 239-248.
3. Alsaleem, F. M., Younis, M. I., & Ouakad, H. M. (2009). On the nonlinear resonances and dynamic pull-in of electrostatically actuated resonators. Journal of Micromechanics and Microengineering, 19(4), 045013.
4. Paul, C. R. (2004). Electromagnetics for engineers. Edition Wiley International, 279.

© 2019 by the authors. Submitted for possible open access publication under the terms and conditions of the Creative Commons Attribution (CC BY) license (http://creativecommons.org/licenses/by/4.0/).
